# Effects of high hydrostatic pressure‐assisted thawing on the physicohemical characteristics of silver pomfret (*Pampus argenteus*)

**DOI:** 10.1002/fsn3.966

**Published:** 2019-04-02

**Authors:** Yan Cui, Xiaoting Xuan, Jiangang Ling, Xiaojun Liao, Huimin Zhang, Haitao Shang, Xudong Lin

**Affiliations:** ^1^ Key Laboratory of Preservation Engineering of Agricultural Products Institute of Agricultural Products Processing Ningbo Academy of Agricultural Sciences Ningbo China; ^2^ College of Food Science and Nutritional Engineering China Agricultural University Beijing China; ^3^ College of Animal Science and Technology Yangzhou University Yangzhou China

**Keywords:** high hydrostatic pressure‐assisted thawing, lipid oxidation, myofibrillar protein, physicochemical characteristics, silver pomfret (*Pampus argenteus*)

## Abstract

Effects of high hydrostatic pressure‐assisted thawing (HPAT, 100, 150, and 200 MPa) on the physicochemical characteristics of silver pomfret were evaluated in comparison with conventional (water immersion thawing, WIT) thawed samples. HPAT significantly decreased the thawing time, as well as the cooking and total losses. The maximum water holding capacity was observed at 100 MPa. Color changed obviously at ≥150 MPa, resulting in a cooked appearance. Samples thawed with HPAT showed better texture quality and lower lipid oxidation. The levels of myofibrillar protein oxidation and surface hydrophobicity increased, while Ca^2+^‐ATPase activities decreased as the pressure increased. The oxidation of myofibrillar protein was significantly decreased at 100 MPa; total sulfhydryl content was 30.85% higher than that of WIT. Overall, 100 MPa is the optimum treatment condition for silver pomfret thawing without negative effects on quality of the product. HPAT can be a potential alternative to produce high‐quality thawed fish.

## 1 INTRODUCTION

Silver pomfret (*Pampus argenteus*), an economically important marine fish species, inhabits the China Sea, Indian Ocean, Oman Sea, as well as North Sea and Arabian Gulf (Archangi, Bazrafshan, Ronagh, Savari, & Abadi, 2013; Davis & Wheeler, 1985; Lan et al., 2018), supporting as a major marine fishery in China in the past several decades (Qin, Xu, Wang, & Shi, 2012; Zhao et al., 2011). Due to its tender meat and high nutritive values, the market demand for silver pomfret is increasing, even though its production declines on account of overfishing and ecological changes (Lan et al., 2018; Wu et al., 2016). However, same as other fishes, silver pomfret is extremely perishable and has a short shelf life owing to its neutral pH, high water activity, high unsaturated fatty acids and free amino acids contents, and exhibition of active autolytic enzymes, making it easy to oxidation, degradation, and decomposition (Lougovois & Kyrana, 2005; Oliveira, Neto, Santos, Ferreira, & Rosenthal, 2017). Freezing is a most preferred and convenient technique for prolonging shelf life and preserving of fish and fish products (Li, Jia, et al., 2014; Li, Wang, Xu, Xing, & Zhou, 2014). Thawing is an inevitable process for consuming and subsequent processing of frozen fish and fish products. Generally, thawing is prevailingly conducted with air, cold water, or warm water, which is time‐consuming and commonly occurs relatively slower than freezing, inducing a significant quality deterioration to frozen food (Eastridge & Bowker, 2011; Li, Jia, et al., 2014). Therefore, a rapid and effective thawing technology that can maintain the “fresh” fish quality is urgently necessary.

In the recent years, nonthermal technologies that can avoid the negative influences of long thawing time and heat on the food qualities (nutritional value, color, texture and flavor etc.), have been widely employed in the field of thawing (Orlowska, LeBail, & Havet, 2014; Rahbari et al., 2018). High hydrostatic pressure (HHP), an effective nonthermal technology for food processing and preservation, has gained increasing interest as a potential thawing tool for its fast thawing rate, low processing temperature, minimal impact on flavor, nutritional, and physicochemical characteristics of food, as well as its ability to sterilize (Oliveira et al., 2017; Park, Ryu, Hong, & Min, 2006; Rouille, Lebail, Ramaswamy, & Leclerc, 2002; Teixeira et al., 2013). Pressure can remarkably influence the ice‐water transitions, and the elevated pressure decreases the phase change temperature of water down to −21°C (minimum) at 210 MPa (LeBail, Chevalier, Mussa, & Ghoul, 2002). The reduction in melting point of water significantly enlarges the temperature gap between the phase change front and the heat source, and thus effectively boosts the heat flux rate, resulting in an accelerated thawing process (LeBail et al., 2002). According to Plank's model, theoretically thawing time of HHP can be half, even one‐fifth of the time of water immersion thawing (WIT) (Zhu et al., 2014), revealing that HHP is a potential alternative for rapid thawing. Zhao, Flores, and Olson (1998) found that thawing of frozen beef with HHP at 210 MPa costed only one twenty‐fourth of the thawing time treated with atmospheric pressure at 3°C, and resulted in similar color and texture to that of conventional thawing. Park et al. (2006) reported that thawing with HHP at <100 MPa could efficiently elevate the physicochemical qualities of frozen pork; the samples treated with HHP showed lower thawing losses and higher water holding capacity (WHC) compared to the atmospheric thawing ones, and the color did not changed up to 100 MPa. Li, Wang, et al. (2014) observed that thawing drip loss significantly reduced with chicken breasts when thawed under HHP. Recently, HHP is becoming more widely applied in the thawing of aquatic products, including Atlantic salmon, aiguillats, scallops, whiting, and cod (Rouille et al., 2002; Schubring, Meyer, Schlüter, Boguslawski, & Knorr, 2003; Zhu et al., 2014). However, few studies have concerned the effects of HHP on the thawing and physicochemical characteristics of the silver pomfret.

This study aims to evaluate, for the first time, the effects of HHP on the thawing and physicochemical characteristics of silver pomfret, comparing with conventional thawing (WIT). In particularly, pressure levels, including 100, 150, 200 MPa, were tested to evaluate their effect on the drip losses, WHC, pH, color, texture, lipid oxidation, protein oxidation, as well as the surface hydrophobicity and Ca^2+^‐ATPase activity, trying to provide a theoretical basis and guidelines for HHP application in thawing of frozen silver pomfret.

## 2 MATERIALS AND METHODS

### 2.1 Materials and chemicals

Fresh silver pomfret (100 ± 10 g average body weight) were purchased and transferred to the laboratory in ice within 1 hr. After cleaning, the fish were rapidly vacuum‐packed and frozen at −80°C, and subsequently stored at −20°C until use. Ca^2+^‐ATPase diagnostic kit was obtained from Nanjing Jiancheng Bioengineering Institute (Nanjing, China). All the chemicals used were of analytical grade obtained from Aladdin Biochemical Technology CO., Ltd (Shanghai, China).

### 2.2 Thawing processes

Fresh silver pomfret samples were set as a blank control (fresh, F). The frozen silver pomfret samples were divided randomly into four groups and subsequently treated by conventional thawing (WIT) or high hydrostatic pressure‐assisted thawing (HPAT). Conventional thawing as a control group was carried out in a 20°C water bath under atmospheric pressure. HPAT was carried out using an HHP unit (CQC2L‐600, Beijing Suyuanzhongtian, China) equipped with a 2 L capacity pressure chamber (100 mm internal diameter and 255 mm height). Distilled water was used as the pressurization medium. Three pressure levels (100, 150 and 200 MPa) were applied for HPAT treatments under 20°C. The rate of pressurization was 3 MPa/s, and pressure was released within 3 s at the end of the holding time. Pressure dwell duration (thawing time, *t*_p_) for HPAT was calculated according to the ratio of the temperature difference (*W*_P_) at HPAT and at atmospheric pressure by the method of Rouille et al. (2002):

$$W_{\text{P}}=\frac{T_{\text{a}}-T_{\text{f}\left(\text{P}\right)}}{T_{\text{a}}-T_{\text{f}\left(\text{Pref}\right)}}[$$

$$t_{\text{p}}=t_{\text{ref}}/W_{\text{P}}$$

where* T*_a_ is the temperature of the thawing medium (^o^C), *T*_f(P)_ is the temperature of fusion of water under pressurization (^o^C), *T*_f(Pref)_is the temperature of fusion of water under reference pressure (atmospheric pressure in this case, ^o^C), and *t*_ref_ is the reference time corresponding to the thawing time at atmospheric pressure (min). In the present case, *T*_a_ was 20°C and *T*_f(Pref)_ was 0°C at atmospheric pressure, so that *W*_P_was around 1.5 at 100 MPa (*T*_f(P)_ was −10°C), 1.75 at 150 MPa (*T*_f(P)_ was −15°C), and 2.0 at 200 MPa (*T*_f(P)_ was −20°C). *t*_ref_ was the thawing time of WIT (20°C) in the present study. The geometric center temperature of WIT samples was detected by a temperature sensor, and the time that the geometric center temperature reached 0–2°C was taken into account as the thawing time (*t*_ref_) (Thanonkaew, Benjakul, Visessanguan, & Decker, 2006). And based on our preliminary experiments, the thawing with HPAT was effectively completed at *t*_ref_/*W*_P_.

All the treatments were performed in triplicate. After thawing, a portion of samples were kept in an ice bath immediately. And the thawing properties, quality indexes were measured within 5 hr. The remaining samples were frozen and stored at −80°C for biochemical assays.

### 2.3 Thawing and cooking loss

Thawing loss of the frozen silver pomfret was determined as the difference between the sample weight before and after thawing, and expressed as a percentage of the initial weight (Xia, Kong, Liu, Diao, & Liu, 2012).

Cooking loss was expressed as the percent in weight differences between the thawed and cooked silver pomfret (80°C, 30 min) based on the weight of the thawed sample.

### 2.4 Water holding capacity (WHC)

Water holding capacity was evaluated using the centrifugation method according to Wang, Xu, Huang, Huang, and Zhou (2014) with minor modifications. The muscle samples (about 10 g) were accurately weighted, and then placed into centrifuge tubes with filter papers at the bottom. After centrifugation at 2,081 *g*, 4°C for 10 min, the samples were removed and reweighed. WHC was expressed as centrifugal loss and computed as the percentage of weight loss to the total weight.

### 2.5 Color analysis

Color measurements of the fish muscle were performed by a CR‐5 colorimeter (Konica Minolta, Tokyo, Japan) using CIE L*a*b* color system. After calibration, the values of L*, a*, and b* were obtained, and donated lightness, redness, and yellowness, respectively. Six different samples were analyzed for each treatment. Three replicates were performed for each measurement. The whiteness index (WI) and total color difference (△*E*), as calculated using the following equations, were also used for evaluation.

$$\text{WI}=100-{\left[{\left(100-L^{*}\right)}^{2}+{\left(a^{*}\right)}^{2}+{\left(b^{*}\right)}^{2}\right]}^{1/2}$$

$$\Delta E={\left[{\left(\Delta L^{*}\right)}^{2}+{\left(\Delta a^{*}\right)}^{2}+{\left(\Delta b^{*}\right)}^{2}\right]}^{1/2}$$

where, △L*, △a*, and △b* are the difference of the L*, a*, and b* values between treated and fresh samples.

### 2.6 Texture profile analysis (TPA)

Hardness, springiness, chewiness, gumminess, and resilience were determined with a TA.XT Plus Texture Analyzer (Surry, UK). The samples were subjected to a two‐cycle compression test using a P/5 cylindrical probe (5 mm diameter) with a 5 g trigger force a test speed 1.0 mm/s to a total deformation 5 mm. The measurements were performed at least 10 times.

### 2.7 pH value

Mince muscles were homogenized with 9 volumes of precooling physiological saline. After standing for 30 min, the filtrates were immediately used for the assays of pH values with a PB‐10 pH‐meter (Sartorius, Gottingen, Germany).

### 2.8 Lipid oxidation

Lipid oxidation of silver pomfret was measured by thiobarbituric acid reacting substances (TBARS) based on the method described in our previous study (Xuan et al., 2018). TBARS values were expressed in mg of malonaldehyde/kg of muscle sample (mg MDA/kg).

### 2.9 Myofibrillar protein preparation

Preparation of myofibrillar protein was conducted using the method described in our previous study (Xuan et al., 2018). The obtained myofibrillar protein solution (MPS) was aliquoted and stored at −80°C before analysis.

### 2.10 Carbonyl content

The carbonyl content of myofibrillar protein was determined by reaction with 2,4‐dinitrohydrazine according to the method reported by Oliver, Ahn, Moerman, Goldstein, and Stadtman (1987). A molar absorption coefficient of 22,000/M/cm was utilized to calculate the carbonyl content. And carbonyl content values of myofibrillar protein were expressed as nmol/mgprot.

### 2.11 Sulfhydryl content

The total sulfhydryl (T‐SH) content of myofibrillar protein was evaluated by Ellman's method (Choi & Park, 2002; Ellman, 1959) with some modifications. About 0.25 ml of MPS was added to 2.5 ml of 0.1 M phosphate buffer (8 M urea, pH 8.0), then incubated with 50 μl 5,5'‐dithio‐bis‐(2‐nitrobenzoic acid) solution (10 mM) at 40°C for 15 min. After that, the absorbance at 412 nm was recorded. A molar absorption coefficient of 13,600/M/cm was utilized to calculate the T‐SH content. And T‐SH values of myofibrillar protein were expressed as nmol/mgprot.

### 2.12 Surface hydrophobicity

The surface hydrophobicity of myofibrillar protein was measured by reaction with bromophenol blue (BPB) based on the method reported by Chelh, Gatellier, and Sante‐Lhoutellier (2006) and expressed as micrograms of bound BPB per milligram of protein (μg/mgprot).

### 2.13 Ca^2+^‐ATPase activity determination

Samples were homogenized with 9 volumes of precooling physiological saline. After centrifugation, the supernatants were diluted and immediately used for the assays of Ca^2+^‐ATPase activity according to the protocol of the corresponding kit. The levels of Ca^2+^‐ATPase activities were expressed in μmol Pi/mgprot/hr.

### 2.14 Statistical analysis

All experiments were performed at least in triplicate. Data were presented as the mean ± standard deviation. The statistics were analyzed using one‐way analysis of variance followed by Duncan's multiple‐range test with a Statistical Package for Social Studies 18.0 software (SPSS Inc., Chicago, IL, USA). A difference of *p *< 0.05 was considered as statistically significant.

## 3 RESULTS AND DISCUSSION

### 3.1 Thawing time

**Figure 1:**
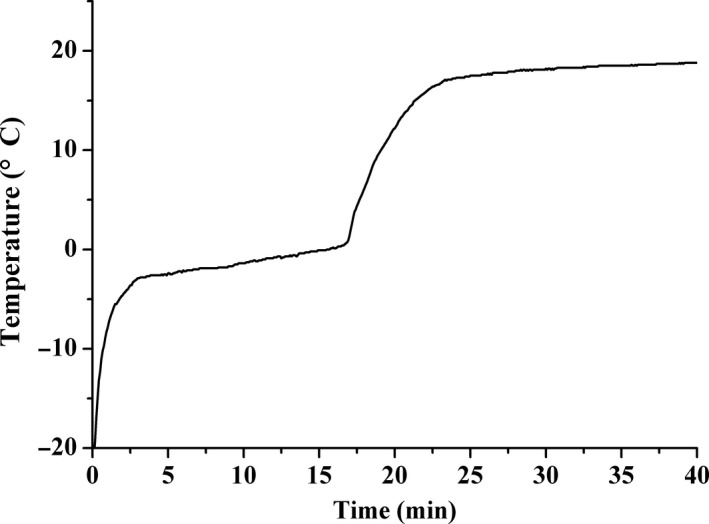
The representative time‐temperature curve for WIT process of frozen silver pomfret

The representative time‐temperature curve for WIT process of frozen silver pomfret was shown in Figure 1. The temperature at the geometric center of sample augmented to reach the melting point and temperature plateau appeared during the WIT process. The duration of the thawing process was 16.67 ± 1.53 min. According to Plank's model, the thawing time is inversely proportional to the temperature difference between the thawing medium and the phase change temperature (Rouille et al., 2002). In the HPAT processes, the phase change temperature is remarkably decreased, and thus the temperature gap between the phase change front and the medium is enlarged during the thawing process, resulting in a greater thawing rate than control (LeBail et al., 2002; Zhu et al., 2014). Therefore, in the HPAT processes, the thawing time could be reduced by the ratio of the temperature difference at HPAT and at WIT (under atmospheric pressure). According to the Plank equation, the thawing time was about 66.67%, 57.14%, and 50.00% of WIT time at medium temperature of 20°C, or 11.11 ± 1.02, 9.52 ± 0.87, and 8.33 ± 0.76 min for HPAT at 100, 150, and 200 MPa, respectively. And under these conditions, thawing processes were all completed. Chourot, Lemaire, Cornier, and LeBail (1995) showed that the actual required thawing time was even shorter than the theoretic value of Plank's model due to the reduction in the latent heat of water under pressure. And the shorter thawing time as the pressure increased may also be related with the pressure‐induced adiabatic heating (pure water at 25°C can increase about 3°C/100 MPa, and high‐fat foods can present temperature increases from 6 to 8.7°C/100 MPa) (Balasubramaniam, Ting, Stewart, & Robbins, 2004).

### 3.2 Effect of HPAT on the thawing, cooking, and total losses

Drip loss is an important parameter impacting the textural and sensory characteristics of thawed fish muscles. In this study, three types of losses, including thawing loss, cooking loss, and total loss, were measured to assess the thaw‐induced quality deterioration. As shown in Table 1, HPAT significantly increased the thawing losses compared to the WIT (*p* < 0.05), which was consistent with the findings reported for cod, Atlantic salmon, and whiting (Chevalier, Le Bail, Chourot, & Chantreau, 1999; Schubring et al., 2003; Zhu, Ramaswamy, & Simpson, 2004). As the pressure elevated, the thawing loss tended to increase, which might be due to the enhanced breaking of the noncovalent bonds of the proteins under pressurization, leading to the higher denaturation of myosin, the principle site of water retention in muscle (Truong, Buckow, Stathopoulos, & Nguyen, 2015; Zare, 2004). However, these findings differ from some published researches. Li, Wang, et al. (2014) observed that under the same high‐pressure levels, the thawing loss was lower in frozen chicken breasts compared to that at atmospheric pressure. Rouille et al. (2002) found that thawing losses of spiny dogfish and scallops were decreased with high‐pressure treatment compared to that of conventional thawing (WIT). These contradictions might result from the diversities in the type of the tested samples, including species, type of proteins, type of muscles, proximate compositions, and texture characteristics. Also, differences in applied process parameters, including freezing and pressurization rates, holding time, can affect treated samples under HPAT.

**Table 1:** Effects of HPAT on the thawing loss, cooking loss, and total loss of silver pomfret

Thawing treatment	Thawing loss (%)	Cooking loss (%)	Total loss (%)
F		26.89 ± 0.16^a^	26.89 ± 0.16^a^
WIT	0.63 ± 0.11^a^	32.77 ± 1.49^c^	33.40 ± 1.38^c^
100 MPa	0.76 ± 0.08^b^	27.05 ± 1.82^a^	27.82 ± 1.57^a^
150 MPa	0.93 ± 0.07^c^	28.61 ± 1.19^ab^	29.54 ± 1.04^b^
200 MPa	1.10 ± 0.09^d^	29.81 ± 1.53^b^	30.91 ± 1.35^b^

Note

Values followed by different superscripts in the same column indicate significant differences (*p* < 0.05).

Excitingly, all the HPAT treatments in this study revealed dramatically decreases in the cooking and total losses (after thawing and cooking) as compared with WIT (*p* < 0.05), revealing that the HPAT would effectively maintain the water retention of fish muscle during cooking process. The lowest drip losses were obtained at 100 MPa treatment, which were almost similar to those of the fresh samples.

### 3.3 Effect of HPAT on water holding capacity

**Figure 2:**
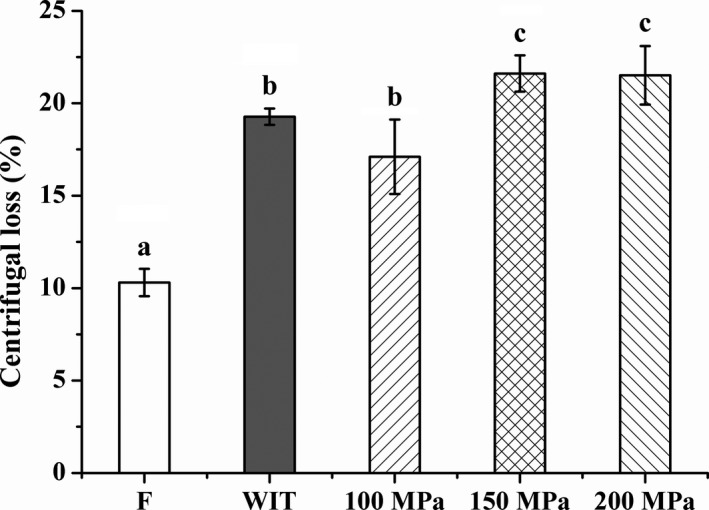
Effects of HPAT on the water holding capacity of silver pomfret. Values followed by different superscripts indicate significant differences (*p* < 0.05)

Water holding capacity, an indicator of the moisture retention property, is an extremely important quality attribute for raw products (Lonergan, Huff‐Lonergan, Wiegand, & Kriese‐Anderson, 2001). As shown in Figure 2, the freezing and thawing process significantly affected the WHC of fish muscles, and the levels of centrifugal loss were all obviously augmented (*p* < 0.05). The maximum WHC was obtained for silver pomfret thawed at 100 MPa. Water losses for raw fish were markedly higher in samples thawed under HPAT treatment at ≥150 MPa compared to that of conventional thawed group (*p* < 0.05). Similar results were found by Tironi, Lebail, and Ilamballerie (2007) that HHP (200 MPa) had a significant reduction in WHC of raw sea bass muscle compared with conventional thawing. Schubring et al. (2003) concluded that compared to WIT, the WHC of raw redfish, salmon, whiting, rainbow trout, and cod fillets was markedly decreased with HPAT treatment (200 MPa). The higher water loss at ≥150 MPa could probably be explained by the denaturation of proteins, which is believed to occur above 150 MPa leading to the modification of the moisture retention capacity (LeBail et al., 2002; Rouille et al., 2002; Tonello, 1997).

### 3.4 Color changes

Color is one of the most important sensory properties of fish in determining their acceptability for consumer's perception (Liu, Zeng, & Sun, 2013). It is known that HHP can cause changes in color of pressurized fish flesh due to the loss of translucency, or the increase in whiteness, resulting in a cooked appearance (Alizadeh, Chapleau, De Lamballerie, & LeBail, 2007; Matser, Stegeman, Kals, & Bartels, 2000; Tironi et al., 2007; Truong et al., 2015). In this study, we reconfirmed this phenomenon. As shown in Table 2, obvious color changes (assessed by very high color differences △*E*) were caused by HPAT. Lightness (L*) and yellowness (b*) showed important increases (*p* < 0.05) in the HPAT‐treated samples. But smaller and uniform changes were observed for redness (a*) (increase). The pressurized silver pomfret muscle became whiter with the increase of L*, which could be visually corroborated. HPAT‐treated samples at ≥150 MPa appeared more white and opaque, resulting in a cooked appearance, which was reconfirmed by the results of WI analysis. As the pressure elevated, the whiteness of fish muscle increased, indicating the greater protein denaturation at higher pressure. It is important to mention that after thawing at 100 MPa, the whiteness of samples was similar to that of the fresh ones, and the fish showed a similar appearance as compared with the fresh silver pomfret without any noticeable visual differences in color.

**Table 2:** Effects of HPAT on the color parameters of silver pomfret muscle

Thawing treatment	L*	a*	b*	WI	△*E*
F	52.94 ± 1.19^a^	−2.58 ± 0.21^a^	5.19 ± 0.96^a^	52.57 ± 1.21^a^	
WIT	55.71 ± 2.71^b^	−1.60 ± 0.27^b^	9.84 ± 0.99^b^	54.58 ± 2.72^b^	3.18 ± 0.48^b^
100 MPa	54.63 ± 1.43^b^	−2.37 ± 0.73^a^	9.92 ± 1.16^b^	53.50 ± 1.49^ab^	2.45 ± 0.56^a^
150 MPa	65.86 ± 1.16^c^	−2.21 ± 0.27^a^	10.58 ± 1.47^bc^	64.14 ± 1.02^c^	11.61 ± 0.64^c^
200 MPa	67.00 ± 1.19^d^	−1.35 ± 0.26^b^	11.21 ± 2.25^c^	65.06 ± 1.29^c^	12.89 ± 0.87^d^

Note

Values followed by different superscripts in the same column indicate significant differences (*p* < 0.05).

### 3.5 Texture profile analysis (TPA)

Texture is an important quality characteristic for fish/fish products and greatly affects consumer acceptability. The TPA parameters of silver pomfret for fresh and thawed samples can be seen in Table 3. HPAT treatments tended to increase the TPA parameters, including hardness, springiness, chewiness, gumminess, and resilience, of silver pomfret compared to those of conventionally thawed samples (WIT). Similar results have been recorded for cod, salmon, haddock, and whiting (Arnaud, de Lamballerie, & Pottier, 2018; Schubring et al., 2003). As the pressure elevated, the hardness and resilience increased significantly from 150 MPa, while the springiness, chewiness, and gumminess increased significantly from 200 MPa as compared to those of WIT (*p* < 0.05). These changes could be correlated to the unfolding of actin and sarcoplasmic proteins, increasing in protein–protein interactions, as well as new hydrogen‐bonded networks formation and cellular damage (Angsupanich & Ledward, 1998; Arnaud et al., 2018; Yagiz et al., 2009; Zare, 2004). It seems that HPAT treatments could modify the texture of the thawed flesh, making it harder and springier, thus preventing the softening of fish flesh during thawing process, which were appreciated during its consumption (Cheret, Delbarre‐Ladrat, de Lamballerie‐Anton, & Verrez‐Bagnis, 2007).

**Table 3:** Effects of HPAT on the TPA parameters of silver pomfret

Thawing treatment	Hardness (g)	Springiness	Chewiness (g)	Gumminess	Resilience (%)
F	1,061.9 ± 124.58^d^	0.76 ± 0.07^a^	633.33 ± 99.41^c^	716.93 ± 105.33^c^	0.42 ± 0.06^a^
WIT	506.06 ± 98.32^a^	0.83 ± 0.03^b^	324.33 ± 67.75^a^	403.75 ± 91.99^a^	0.41 ± 0.07^a^
100 MPa	568.94 ± 106.87^ab^	0.83 ± 0.02^b^	329.72 ± 75.67^a^	400.82 ± 91.13^a^	0.43 ± 0.03^a^
150 MPa	673.19 ± 152.66^bc^	0.85 ± 0.03^b^	365.05 ± 72.10^a^	421.41 ± 85.07^a^	0.47 ± 0.04^b^
200 MPa	740.66 ± 151.59^c^	0.89 ± 0.03^c^	450.47 ± 97.50^b^	509.64 ± 103.94^b^	0.47 ± 0.03^b^

Note

Values followed by different superscripts in the same column indicate significant differences (*p* < 0.05).

### 3.6 pH changes

**Figure 3:**
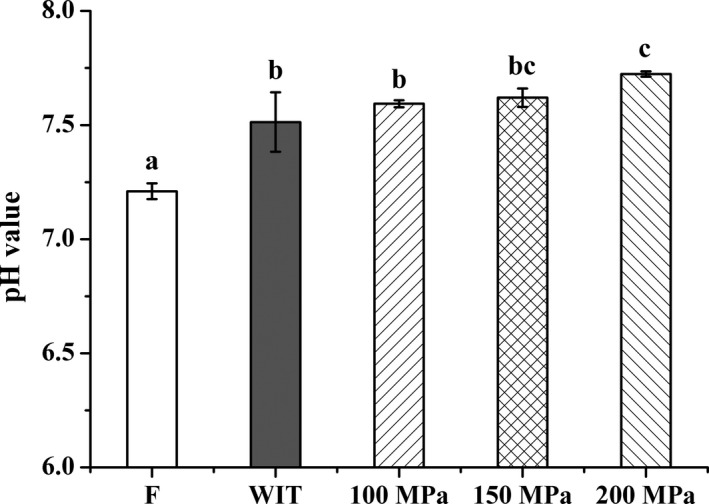
Effects of HPAT on the pH values of silver pomfret. Values followed by different superscripts indicate significant differences (*p* < 0.05)

pH was 7.21 ± 0.03 for the fresh silver pomfret samples. The pH value of silver pomfret thawed with HPAT was found to be dependent on pressure levels (Figure 3). As the pressure increased, the pH of HPAT‐treated samples increased, and showed a significant increase at 200 MPa relative to the conventionally thawed samples (*p* < 0.05).

The increase in pH value with the elevation of pressure is consistent with the findings obtained with oysters by Cruz‐Romero and co‐authors (Cruz‐Romero, Kerry, & Kelly, 2008; Cruz‐Romero, Smiddy, Hill, Kerry, & Kelly, 2004). The pH represents a measure of the overall quality and freshness for seafood products, and the elevated pH observed with the increasing pressure could be related to the protein denaturation, resulting in a decline of available acidic groups with the protein unfold (Angsupanich & Ledward, 1998; Yi et al., 2013). In addition, the ionization of these acidic groups and self‐ionization of water are also favored by pressurization (Ramirez‐Suarez & Morrissey, 2006; Schubring et al., 2003; Yamamoto, Yoshida, Morita, & Yasui, 1994).

### 3.7 Lipid oxidation

**Figure 4:**
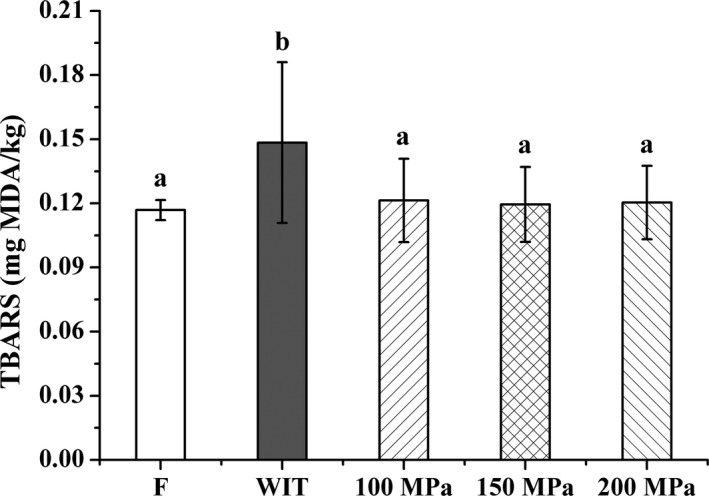
Effects of HPAT on the lipid oxidation of silver pomfret. Values followed by different superscripts indicate significant differences (*p* < 0.05)

Lipid oxidation, one of the major causes of quality deterioration in fish, is of great concern to fish industry because it leads to undesirable rancidity, flavor, and color changes, reducing the consumer acceptability of fish muscle especially in fatty fish (Truong et al., 2015; Venugopal, 2006). Figure 4 displays the changes in TBARS levels of silver pomfret samples thawed by HPAT. The mean value of TBARS in fresh sample was 0.11 ± 0.00 mg MDA/kg and found to be remarkably enhanced by thawed under conventional process (*p* < 0.05). A slight increase in TBARS values was observed in all HPAT‐treated samples, but no significant change was recorded when compared with the fresh one, which was in accordance with the findings obtained with cod muscle (Angsupanich & Ledward, 1998). However, studies have also been reported that lipid oxidation was accelerated in many aquatic products, including black tiger shrimp, salmon, and razor clam after pressurization (Arnaud et al., 2018; Kaur, Rao, & Nema, 2016; Xuan et al., 2018), indicating that effects of HHP on lipid oxidation of muscles varied vastly on the species, type of muscles, applied pressure level and holding time.

### 3.8 Protein oxidation

**Figure 5:**
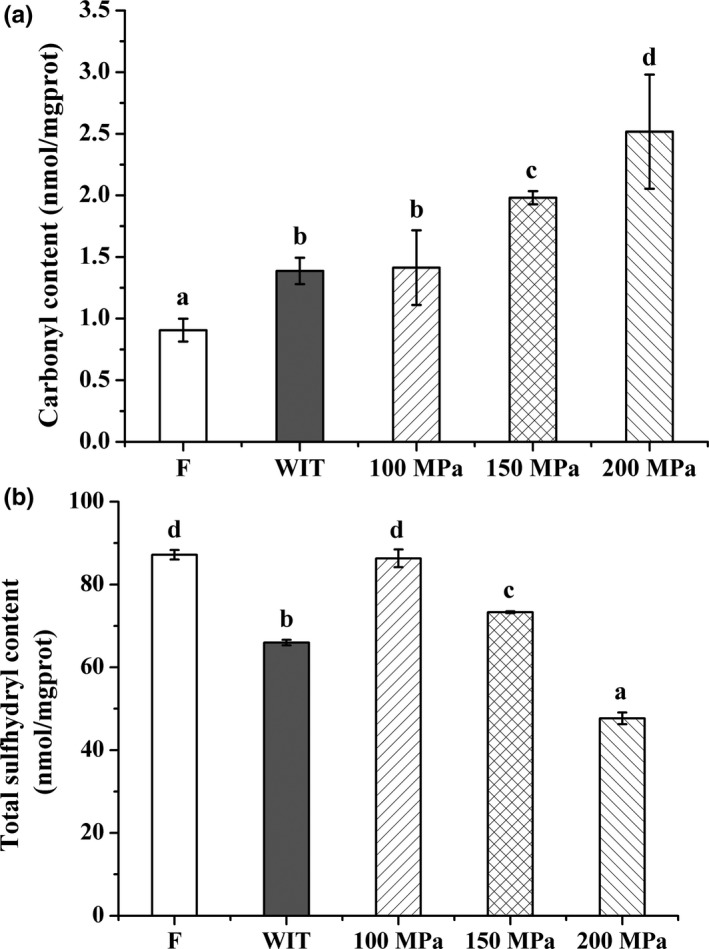
Effects of HPAT on the (A) carbonyl and (B) total sulfhydryl contents of myofibrillar protein in silver pomfret. Values followed by different superscripts in each testing parameter indicate significant differences (*p* < 0.05)

It is assumed that the HHP processing of fish muscles accelerates protein oxidation (Oliveira et al., 2017). Proteins are susceptible to attack by free radicals and carbonylation is one of the most significant changes in oxidized proteins (Jia, Nirasawa, Ji, Luo, & Liu, 2018; Zhang, Fang, Hao, & Zhang, 2018). During high‐pressure processing, myofibrillar protein (the main structural protein constituting approximately 40%–60% of fish muscle proteins) undergoes a range of changes, such as denaturation, degradation, aggregation, and oxidation which results in a wide range of modifications, especially the formation of carbonyl compounds (Arnaud et al., 2018; Lund & Baron, 2010; Shao et al., 2018; Truong et al., 2015). In this study, we reconfirmed this phenomenon. The carbonyl content of myofibrillar protein in silver pomfret muscle remarkably (*p* < 0.05) increased with the treatment of HPAT or WIT (Figure 5A), indicating an obvious protein oxidation occurred during the thawing process. The carbonyl content of silver pomfret treated with HPAT was found to be dependent on the pressure levels. As the pressure increased, the carbonyl content of HPAT‐treated samples remarkably increased and showed an obvious increase with the treatment at ≥150 MPa relative to the conventionally thawed samples (*p* < 0.05). The increased carbonyl contents under pressure can be attributed to the promotional formation of free radicals acting as initiation of protein oxidation reaction related with the conformational changes in myofibrillar protein, leading to aggregation, formation of off flavors, and reduction in functionality and nutritional value (Bolumar, Andersen, & Orlien, 2014; Guyon, Meynier, & de Lamballerie, 2016; Oliveira et al., 2017).

As shown in Figure 5B, the content of total sulfhydryl, one of the most used markers for protein oxidation, was also determined. The results revealed that sulfhydryl groups, the most reactive functional group in proteins, are easily oxidized to disulfide bond during the freeze‐thawing process, causing a decrease in T‐SH content. The initial T‐SH level of fresh muscle was 87.18 ± 1.16 nmol/mgprot. After thawing, the T‐SH content of the WIT‐treated samples significantly decreased to 65.97 ± 0.66 nmol/mgprot (*p* < 0.05), demonstrating a significant protein oxidation. In accordance with the literature (Zhang, Yang, Tang, Chen, & You, 2015), prominent decreases in T‐SH levels were observed after HPAT treatments, which were negatively correlated with the results of carbonyls formation. Hsu, Jyh‐Sheng, Yu, and Jao (2007) and Zhou et al. (2014) reported that the T‐SH values of actomyosin from threadfin bream and tilapia also decreased sharply with the increase in pressure, which might be related to the distance declines between sulfhydryl groups under pressure, leading to the formation of disulfide bonds (Cheftel, 1995; Zhou et al., 2014). However, the samples treated with 100 and 150 MPa were in comparatively good condition. The T‐SH content was 86.32 ± 2.14 and 73.31 ± 0.22 nmol/mgprot, respectively, which were dramatically higher than that of the control samples (WIT, *p* < 0.05). It is important to mention that after thawing under 100 MPa, the level of T‐SH was similar to that of the fresh one. All the results above indicated that compared to WIT, the HPAT at 100 MPa could markedly alleviate the protein oxidation during thawing. The lower carbonyl content and higher T‐SH content in the myofibrillar protein of silver pomfret with 100 MPa might be one of the reasons contributing to lower cooking loss and total loss (Table 1).

### 3.9 Surface hydrophobicity

**Figure 6:**
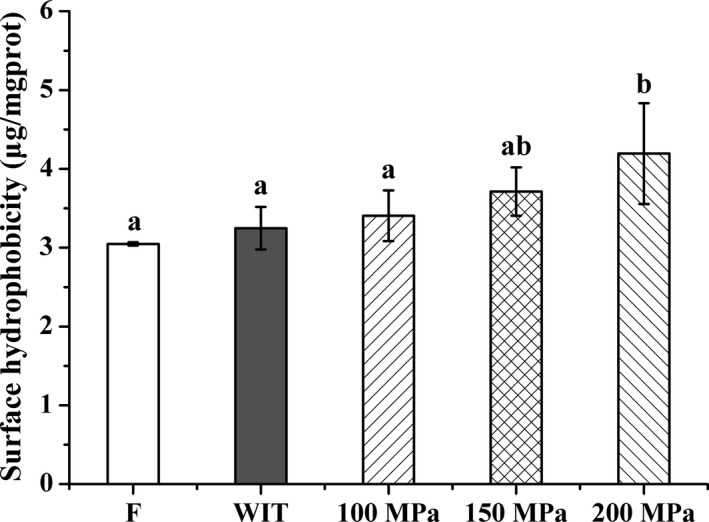
Effects of HPAT on the surface hydrophobicity of myofibrillar protein in silver pomfret. Values followed by different superscripts indicate significant differences (*p* < 0.05)

Changes in surface hydrophobicity of myofibrillar protein from silver pomfret muscle induced by HPAT treatment are depicted in Figure 6. Similar with the previous observations (Chapleau, Mangavel, Compoint, & Lamballerie‐Anton, 2004; Zhang et al., 2015), the levels of surface hydrophobicity elevated slowly from 0.1 to 150 MPa, and showed a sharp elevation at 200 MPa when compared to the fresh samples (*p* < 0.05), suggesting that the pressure level was significantly positive with the surface hydrophobicity levels.

Surface hydrophobicity changes indicated the conformational changes in protein structure. Chapleau et al. (2004) found that pressure‐induced increase in hydrophobicity in proteins might be resulted from modifications of protein structure leading to the appearance of new hydrophobic sites. Ko, Jao, and Hsu (2003) reported that hydrophobic interactions were strengthened due to the exposure of amino acid residues to the protein surface under pressure. The pressure‐induced hydrophobicity might be also related to the unfolding of proteins and exposure of buried hydrophobic residues (Alvarez, Ramaswamy, & Ismail, 2008). The results of our study revealed that thawing of silver pomfret with HPAT ≤150 MPa had no obvious effect over the entire surface of the myofibrillar protein molecule. Levels of surface hydrophobicity were remained nearly the same as that of the fresh samples.

### 3.10 Ca^2+^‐ATPase activity

**Figure 7:**
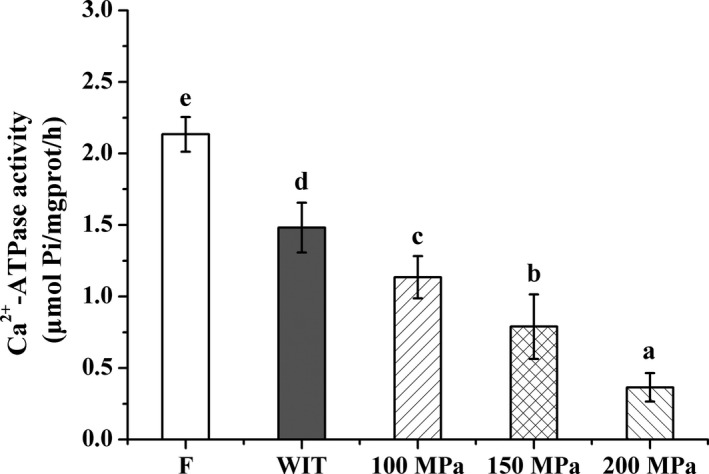
Effects of HPAT on the Ca^2+^‐ATPase activity of silver pomfret. Values followed by different superscripts indicate significant differences (*p* < 0.05)

Ca^2+^‐ATPase activity, a good indicator of protein structural alterations, is closely related to the integrity of myosin; a reduction in Ca^2+^‐ATPase sensitively reveals the loss in Ca^2+^‐ATPase regulation of tropomyosin, which presumably causes the conformational changes (Benjakul, Seymour, Morrissey, & An, 1997; Benjakul, Visessanguan, Thongkaew, & Tanaka, 2003). Changes in Ca^2+^‐ATPase activity of silver pomfret with or without pressure during thawing are shown in Figure 7. The activities of Ca^2+^‐ATPase were distinctly reduced in HPAT‐treated samples compared to that obtained from fresh samples (*p* < 0.05). And the loss of Ca^2+^‐ATPase activity with the elevating pressure was remarkable in all HPAT‐treated samples (*p* < 0.05). Similar changes were also found by other researchers. Cheng, Sun, Zhu, and Zhang (2017) revealed that the activity of Ca^2+^‐ATPase in natural actomyosin from prawn (*Metapenaeus ensis*) was markedly decreased, and activity remained only 42% after 100 MPa treatment. Zhou et al. (2014) reported that the Ca^2+^‐ATPase activity of natural actomyosin in threadfin bream muscle sharply lost, and no activity was observed after pressurization under pressure (≥200 MPa). Ko et al. (2003) noted that the Ca^2+^‐ATPase in tilapia myosin was susceptible to pressure; the activities declined dramatically when treated ≥100 MPa and remained only 43%, 35%, and 21% by 100, 150, and 200 MPa treatment for 10 min, respectively. Our previous study also showed the adverse effects of pressure on the activity of Ca^2+^‐ATPase of razor clam (Xuan et al., 2018). In this study, approximately 53.21%, 37.02%, and 17.11% activity remained after HPAT at 100, 150, and 200 MPa, respectively. The patterns of Ca^2+^‐ATPase, which is an indicator of myosin integrity, highlighted the complex nature of the physical interactions and chemical reactions that take place during the HPAT process of silver pomfret. Generally, the activity of Ca^2+^‐ATPase depends on the conformational structure of myosin (Wang et al., 2017). It is closely associated with the oxidation of SH groups in myosin, especially in myosin globular head, causing inactivation of Ca^2+^‐ATPase (Kittiphattanabawon, Benjakul, Visessanguan, & Shahidi, 2012; Wang et al., 2017). So, the pronounced loss of T‐SH after HPAT treatments (Figure 5B) would explain the corresponding activity reduction in Ca^2+^‐ATPase. In addition, the loss of Ca^2+^‐ATPase activity under pressure might also be attributed to the conformational changes in the myosin subfragment‐1, which unfolded, exposing hydrophobic groups, with a concomitant aggregation of these molecules (Cheng et al., 2017; Yamamoto, Hayashi, & Yasui, 1993; Zhou et al., 2014).

## 4 CONCLUSIONS

The present study demonstrated that HHP was applicable to the thawing process of frozen silver pomfret. Although adverse effects exist, especially on muscle color and Ca^2+^‐ATPase activity, HPAT showed encouraging results in comparison with conventional thawing (WIT). Time saving is the most evident advantage of HPAT; the thawing time can be reduced by approximately 33.33%, 42.86%, and 50.00% at 100, 150, and 200 MPa, respectively. Samples maintained under pressure showed an increase in hardness, springiness, chewiness, gumminess, and resilience, as well as a reduction in cooking loss and TBARS formation. 100 MPa is the optimum for silver pomfret thawing and maintaining its quality. The cooking and total loss (after thawing and cooking) were reduced by 17.44% and 16.71%, respectively, at 100 MPa relative to the conventionally thawed samples. It is important to mention that, HPAT at 100 MPa could also markedly alleviate the protein oxidation during the thawing process; T‐SH content was 30.85% higher than that of WIT. In addition, silver pomfret thawed at 100 MPa showed similar appearance, without any noticeable visual differences in color as compared with the fresh silver pomfret. These results suggest that HPAT at 100 MPa could alleviate the quality deterioration compared to that from conventional thawing method, thus making it a promising alternative to the silver pomfret thawing.

## CONFLICT OF INTEREST

The authors declare no conflict of interest.

## ETHICAL STATEMENT

This article does not contain any studies with human or animal subjects performed by any of the authors.
